# Living Situation Affects Adherence to Combination Antiretroviral Therapy in HIV-Infected Adolescents in Rwanda: A Qualitative Study

**DOI:** 10.1371/journal.pone.0060073

**Published:** 2013-04-03

**Authors:** Philippe R. Mutwa, Jennifer Ilo Van Nuil, Brenda Asiimwe-Kateera, Evelyne Kestelyn, Joseph Vyankandondera, Robert Pool, John Ruhirimbura, Chantal Kanakuze, Peter Reiss, Sibyl Geelen, Janneke van de Wijgert, Kimberly R. Boer

**Affiliations:** 1 Kigali University Teaching Hospital (KUTH)/Department of Pediatrics, Kigali, Rwanda; 2 Academic Medical Centre, Department of Global Health/Amsterdam Institute for Global Health and Development (AMC-AIGHD), Kigali, Rwanda and Amsterdam, The Netherlands; 3 Wayne State University, Department of Anthropology, Detroit, Michigan, United States of America; 4 University of Liverpool, Institute of Infection and Global Health, Liverpool, United Kingdom; 5 Projet Rinda Ubuzima, Kigali, Rwanda; 6 Utrecht University Children’s Hospital/University Medical Centre Utrecht (UMCU), Utrecht, The Netherlands; 7 Royal Tropical Institute (KIT), Biomedical Research, Epidemiology Unit, Amsterdam, The Netherlands; 8 Treatment and Research on HIV/AIDS Centre (TRAC-plus)/Outpatients Clinic, Kigali, Rwanda; 9 Belgian Development Agency, Kigali, Rwanda; 10 Centre for Social Science and Global Health, University of Amsterdam, Amsterdam, The Netherlands; Africa Centre for Health and Population Studies - University of KwaZulu-Natal, South Africa

## Abstract

**Introduction:**

Adherence to combination antiretroviral therapy (cART) is vital for HIV-infected adolescents for survival and quality of life. However, this age group faces many challenges to remain adherent. We used multiple data sources (role-play, focus group discussions (FGD), and in-depth interviews (IDI)) to better understand adherence barriers for Rwandan adolescents. Forty-two HIV positive adolescents (ages 12–21) and a selection of their primary caregivers were interviewed. All were perinatally-infected and received (cART) for ≥12 months. Topics discussed during FGDs and IDIs included learning HIV status, disclosure and stigma, care and treatment issues, cART adherence barriers.

**Results:**

Median age was 17 years, 45% female, 45% orphaned, and 48% in boarding schools. We identified three overarching but inter-related themes that appeared to influence adherence. Stigma, perceived and experienced, and inadvertent disclosure of HIV status hampered adolescents from obtaining and taking their drugs, attending clinic visits, carrying their cARTs with them in public. The second major theme was the need for better support, in particular for adolescents with different living situations, (orphanages, foster-care, and boarding schools). Lack of privacy to keep and take medication came out as major barrier for adolescents living in congested households, as well the institutionalization of boarding schools where privacy is almost non-existent. The third important theme was the desire to be ‘normal’ and not be recognized as an HIV-infected individual, and to have a normal life not perturbed by taking a regimen of medications or being forced to disclose where others would treat them differently.

**Conclusions:**

We propose better management of HIV-infected adolescents integrated into boarding school, orphanages, and foster care; training of school-faculty on how to support students and allow them privacy for taking their medications. To provide better care and support, HIV programs should stimulate caregivers of HIV-infected adolescents to join them for their clinic visits.

## Introduction

In Rwanda, at the end of 2011 about 26,486 children were living with HIV, with 7,356 on cART [1]. Fortunately, with the increased availability of cART more children born with HIV survive into adolescence and adulthood [2], [3]. The focus of treatment has thereby changed from management of a severe debilitating disease to more long-term care with challenges such as maintaining treatment success, management of chronic co-morbidities, supporting adherence to life-long therapy and prevention of HIV drug resistance [4]–[6].

Studies in the region have shown that treatment failure in children can be as high as 38% after both short and medium long term evaluations [7]–[9]. Poor adherence is a driving cause of treatment failure, HIV drug resistance, disease progression [10]–[12] and HIV transmission [13], [14], whereas 95% cART adherence is associated with reduced morbidity and mortality [15]–[17]. Therefore adherence should be one of the main concerns when providing cART, especially in resource limited settings where second and third line therapies are not available or generally too expensive.

There are different barriers to adherence in children, and particularly adolescents, than in adults. Besides adolescence being a turbulent and vulnerable period in life with many physical and emotional changes, other factor, such as being orphaned, or school conditions may pose extra challenges [18]. Adherence seems to vary within studies, populations and countries; a recent study in Rwanda has reported only 45% of children (orphans and non-orphans) taking all of their prescribed cART medication in the previous month [19] although some studies report significantly higher adherence rates in pediatric populations [20], [21]. Therefore to better understand cART adherence barriers and successes in adolescents in Rwanda, we conducted a qualitative study with perinatally HIV-infected adolescents and their primary caregivers.

## Methods

### Study Setting and Participants

Adolescents were recruited from the HIV outpatient clinic of the Center for Treatment and Research on AIDS, Tuberculosis and Malaria (TRACplus), a National center for infection control and prevention in Rwanda. This clinic was chosen for the following reasons; it is one of the major HIV-pediatric clinics in Rwanda and the first clinic to provide cART in children since 2004. Additionally, adolescents at this clinic have regular group meetings as part of routine care. At the time of the data collection for the study, the clinic provided care to 600 HIV-infected children and adolescents of whom 444 were receiving cART; 384 out of these 444 were on cART for 12 months or longer and 179 out of these 384 were ≥12 years.

Inclusion criteria for the study were: 12–21 years of age, HIV infection, on cART for ≥12 months, and a planned clinic visit during the 2 months of the study period. Study participants included also a selection of primary caregivers. The parents/caregivers were selected based on their availability, since we planned one FGD for the parents; of the 18 parents who agreed to participate, 10 showed up during for the discussions, including 6 women and 4 men.

Eligible adolescents and their primary caregivers were contacted by study staff, the objectives and study procedures were explained to them. Adolescents were excluded if they or their primary caregivers were unwilling to participate or unable to attend the clinic during the study period. All adolescents older than 18 years provided written informed consent to participate in the study, and all adolescents between 12 and 18 years were asked assent. Parents or legal guardians provided the written informed consent on behalf of the child below 18 years of old as advised by the Rwandan National Ethic Committee.

### Study Procedures

Study methods included role-playing sessions, focus group discussions (FGDs) and in-depth interviews (IDIs) with adolescents and their caregivers. The data were collected by two nurses and the process was supervised by a doctor. All three received prior training during a five-day skills-based training on moderating FGDs and IDIs in which the FGD scripts and IDIs were developed (Table 1). Each topic discussion began with a role-play session to elicit topics and allow adolescents to feel more comfortable with the topics. After each role-play, they were asked to comment and ask questions related to the topics during the FGD.

**Table 1:** Topics for FGD and IDI - Adolescents and primary caregivers.

Topics for FGD and IDI with adolescents	Topics for FGD with primary caregivers
Learning HIV status	HIV disclosure
Disclosure to others (and leaning HIV status) and stigma	
Issues related to care and treatment such as difficult in taking medication,medication change, experiencing side effect, clinical care services providedby health providers etc…	Issues related to care and treatment
Issues related to adherence	Issues related to adherence
Health-seeking behavior, and social support	Health-seeking behavior, and social support

Three mixed gender FGDs and 8 IDIs for adolescents, and one FGD with 10 primary caregivers, were conducted from October to November 2010. The interviews/discussions were conducted over 2 or 3 days from 8 to 4 PM each day outside of the clinical services area in a recreational facility to ensure confidentiality and comfort. Adolescents used nicknames instead of their real name during the discussions. The interviews were digitally recorded in Kinyarwanda, transcribed and translated from Kinyarwanda into English, and uploaded into ATLAS.ti for analysis. All recordings and translations were stored in a secure locked place. The Rwandan National Ethics Committee and National AIDS Control Program approved this research and annexes including informed consent written in English with translated into Kinyarwanda.

### Analytic Approach

The data were coded using framework analysis as described by Krueger [22], during which process a list of categories were derived from multiple readings of the data. Each core category led to the development of a code. The codes were refined, revised, specified and elaborated in successive returns to the data. More than one person coded the same section of the data to ensure a level of consistency for the coding procedure.

Thematic analysis was then used to further understand challenges related to adherence in adolescents. These themes enabled us to group different codes together and to provide better insight into the adherence barriers, strategies and resolutions children use to adhere to cART and also dilemmas and sources of adherence support.

## Results

### Baseline Characteristics

**Figure 1:**
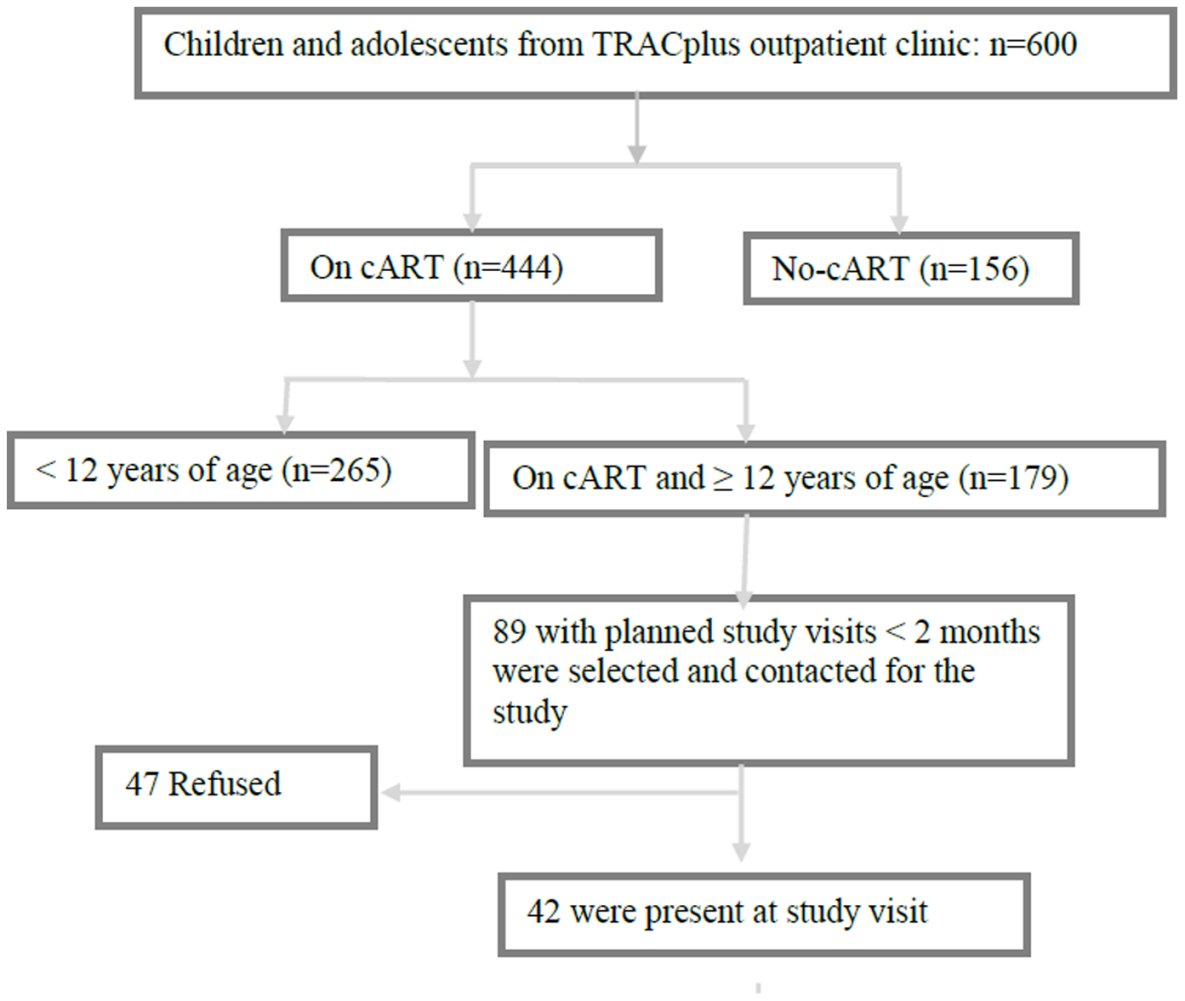
Flowchart summarizing patient inclusion from TRACplus Healthcare center.

There were 179 eligible adolescents ≥12 years of age. Eighty-nine of these adolescents had planned visits to the TRACplus clinic in the following 2 months for their routine clinical care and therefore were contacted to join the study. Forty-seven adolescents were unavailable for the study visit or refused participation (either adolescent or caregiver refused); leaving 42 adolescents in the study representing 47% of the eligible adolescents, and 23% of all adolescents treated in the clinic and on cART for more than 12 months (Figure 1). The parents/caregivers included 6 biological parents, 2 other family members and 2 foster care guardians. Median age for adolescents was 17 years (ranging from 12 to 21), 45% were female, and 45% orphaned (Table 2). All adolescents were reported to be perinatally HIV infected. Seven inter-related topics associated with cART adherence were elicited from the data: the desire to be healthy, HIV-stigma, disclosure and acceptance of HIV status, availability and lack of social support, isolation and depression, cART medication regimen demands, and challenges related to living in a boarding school. Although the topics were discussed separately, they were often related and experienced simultaneously.

**Table 2:** Baseline Characteristics.

Demographics N = 42	n (%)
Gender, female	19 (45%)
Age, median years (range)	17 (12–21)
Orphaned, (for both parents)	19 (45%)
Live with one parent, in orphanages or foster families	31 (74%)
Living in boarding school	30 (71%)
**Reason for HIV testing**	
Illness	14(33%)
Illness or death of siblings	8(19%)
Illness or death of parents	20(48%)
**cART regimen**	
Median (IQR) cART duration, years.	5 (3–6)
AZT or ABC or D4T +3TC+ NVP	26 (62%)
AZT +3TC+EFV	13(31%)
ABC+3TC+ LPV/r	3(7%)

### Desire to be Healthy

The most frequently cited theme was in reference to the health effects of cART, including effects that improved adherence (e.g. feeling better and reducing infections) and those that diminished adherence (e.g. side-effects and misconceptions of the medication). Many adolescents discussed their poor health before initiating medication and how their health improved and they were able to resume usual activities after initiation of treatment. The medication gave them not only physical health (such as making them grow or gain weight (section 1, quote 1), but also hope and the ability to lead a “normal” life. The adolescents used the words “normal” and “healthy” interchangeably throughout the FGDs (section 1, quote 2). Adolescents emphasized the wish to continue living as their HIV uninfected peers. The personal experiences of health improvement, including evidence of increased CD4 levels, encouraged them to remain adherent to their medication schedules (section 1, quote 3).

### Section 1: Quotes Regarding the Desire to be Healthy

*“…before starting medication, I used to eat but I was not putting on weight, but after taking medication…there was improvement in my general health.”* Adolescent FG2

*“Before you start medication you are in an abnormal state, but after starting the medication your CD4 count increases, then the HIV virus is weakened, and then you look as if your body has returned to a normal state.”* Adolescent FG2

*“I stopped taking my medication and my CD4 dropped to only 37 ….when I restarted my medication, my CD4 increased up to 200 and I felt that I am capable of changing the course of my life and doing things just like HIV uninfected people…. ….”* Adolescent FG1

### Stigma and Desire for Privacy

Stigma, both perceived and experienced, hampered adolescents from both obtaining and taking their drugs. Adolescents described how they would avoid going to the clinic to obtain their drugs because they did not want community members to see them. At times, family members who were asked to pick up the medication refused, for fear of being seen and labeled as living with HIV (section 2, quote 1).

A related challenge for many adolescents was a lack of privacy. This included lacking a private place to keep and take their medication; often living in congested households, boarding schools and foster care. Stigma played an important role in the adolescents’ adherence even within their own homes; they did not want their siblings, friends, and others to see them take their medication (section 2, quote 2). In over half of the discussions regarding stigma, adolescents had the intention to be adherent but due to lack of privacy, they skipped their dose or failed to obtain refills from the clinic to avoid being seen. Stigma was not just perceived, but also experienced, with people not wanting to share plates or cups and some people making derogatory comments (section 2, quote 3). Privacy was a particular challenge for adolescents living in boarding schools. They had trouble finding a place to keep and take their medication; they felt that they had to hide their pill bottles from both faculty and other students. They spoke about how other students would sometimes rummage through their belongings thereby making it difficult to keep the medication hidden (section 2, quote 4).

### Section 2: Quotes Regarding Perceived and Experienced Stigma and Lack of Privacy

*“… during that time I was seriously ill so that I was unable to go to pick-up my medication. My sister hesitated in regard to collecting it on my behalf, because people could brand her as HIV positive.”* Adolescent FG2

*“I think that the fact of not living with your own parents is a substantial obstacle. For example, when you live with an aunt or uncle, most of the time, their children may not be aware of your problem [HIV-status] and it becomes a problem for you to take your medication when you are sharing the same bedroom or bag.”* Adolescent FG2

*“I experienced stigma at home and in the boarding school. I revealed [my HIV-status] to my boyfriend …and also told my brother…he divulged it to his wife, who, in the end, made it public. Whenever I passed by they referred to me as dead person walking.”* Adolescent FG1

*“When I was going to boarding school, they gave me drugs for three months and I kept them in my bag. As each one had his/her own bed, I had to cover myself with the sheet to swallow the tablets…however, someone tried to steal my stuff by cutting open my bag…where they found my medication and scattered it on my bed. When other students came back, they asked to whom the drugs belonged and I said I didn’t know. So you can understand that keeping medication in a dormitory is risky.”* Adolescent FG2.

### Disclosure of HIV Status

Directly related to stigma were challenges surrounding disclosure and acceptance of one’s HIV status. Disclosure is directly related to both perceived and experienced stigma; many of the discussions regarding the fear of being stigmatized were linked with issues of non-disclosure in the community or boarding school. Several adolescents and parents discussed the difficulties in having one HIV-infected child and the others healthy. They also described how difficult it is to have the child remain adherent when most of the siblings were unaware of the HIV status. Not sharing one’s HIV status with family members, especially other children who were not HIV-infected was a theme that came up several times from both caregivers and adolescents.

All adolescents in the study were reported being infected with HIV perinatally, which brings a unique set of challenges, especially regarding disclosure. Unlike most adult populations, adolescents face not only issues of disclosing to others, but also coming to terms when they are informed of their own HIV status. In some instances, the adolescent’s HIV status was kept hidden even after s/he began cART and s/he did not exactly know what the medication was for. When the doctor or caregiver disclosed their status, it often helped the participants to realize the importance of adherence (section 3, quote 1). In a way, cART became a route through which the adolescent learned about his/her status and the importance of the medication. It was discussed at length that finding out their own HIV status led to adolescents expressing confusion or anger towards their parents. This anger in some cases also led to the adolescent being less adherent, sometimes in an attempt to punish their parents and other times in coming to terms or due to confusion about why this happened to them and not to others (sometimes their own siblings). (section 3, quote 2 to 3).

### Section 3: Quotes Regarding Disclosure of HIV Status

“After finding out my HIV status, I was able to take *the medication regularly and on time. So I feel that in order for medication to be taken properly one must be told thoroughly why he is taking it.”* Adolescent FG2

*“There is a mother with a child infected by HIV, every time this child is admitted to the hospital she says to her mother, ‘all this is because of you, you waste your time giving me soup, what kind of love are you showing me? All of this is because of you’. That mother cries as the child tells her, ‘why did you give birth to me while you knew you were infected?’.”* Caregivers FGD

*“We want to talk to our parents because they are the cause of our illness…only then can they truly understand our concerns how much it hurts to be born as an HIV positive child…. They even beat us, when we fail to take our medication while they are the cause of our HIV infection.”* Adolescent FG3

### Acceptance, Isolation and Depression

The adolescents discussed that non-acceptance of their HIV status, could lead to feelings of depression and isolation. In turn, depression can prevent people from taking their medication (section 4, quote 1). Both in the adolescent FGDs and caregivers FGD, depression and isolation were often related to anger and confusion, with adolescents questioning why he or she became infected while his or her siblings were not (section 4, quote 2). Adolescents discussed that once they accepted their disease, and had the will to live they were more adherent (section 4, quote 3 and 4). When depressed and isolated, they were less motivated to take their cART. This links strongly with social support and their living situations. When living with one’s own family/parents, adolescents generally found more support. But some adolescents had difficulty sharing with their own families, including their siblings. Parents described this as very difficult situation (section 4, quote 5).

Alcohol use was not a common problem, but mentioned in relation to acceptance of status and depression and isolation. The adolescents discussed how if they did not accept their status, they might resort to casual substance use, which was also associated with forgetting to take cART.

### Social Support and Living Situations

There were some short discussions regarding religious support and practices, such as fasting and strategies to overcome religious barriers to cART adherence. Some adolescents discussed religious healing or cures; however in general they agreed that, although religion was important, they should remain on their medication (section 4, quote 6); with only one adolescent stating that she had faith that God would cure AIDS.

However, living situations caused the most issue for cART adherence for these adolescents. When adolescents were living with their own family, they felt their family members, including parents and siblings, generally provide a supportive environment, often reminding them to collect and take their medication. However, 19 out of 42 were orphans taken care of by orphanages or foster families and 30 out of 42 were students living in boarding schools. Some of the orphans also were living in boarding schools. According to the adolescents those living in foster families may not have the same level of support from their foster parents as they would from their own parents (section 4, quote 7). Some found that their foster parents cared less about them taking their medication, some found little support at home, and others even found that they were discriminated against in the family and that stigma in their own home led them to take their medication less.

For the many adolescents living with HIV who live in boarding schools cART adherence was a complex situation. They found that the school did not accommodate their cART needs, making it difficult for them to access their medication. Adolescents reported that some teachers knew about their status but were unsupportive and did not assist them with adherence. The adolescents spoke about how some schools requiring an HIV test before admission as a way to “enable them to put you in a given category” in order to provide better healthcare if the student falls ill. Many adolescents voiced their concerns regarding privacy, disclosure and subsequent stigma in boarding schools. One adolescent commented, “Stigma is noticed but it only takes place when other students know your HIV status, then they start to reject you.” The majority of the problems were associated with privacy for storing and taking medications within the boarding school. Further, students sometimes had conflicts with their medication schedule and their class schedule: the teachers would not allow them to leave the class so that they are forced to take their medication in front of other students and had to avoid inadvertently disclosing their status (section 5, quote 1). These situations led to students becoming uncomfortable, isolated, and non-adherent. There was also talk of supportive boarding school staff; adolescents spoke about how administrators would keep the medication in their offices to increase privacy and some boarding school teachers would assist them in storing and taking their medication. One adolescent reported that the activities coordinator of the school reached out and established trust, thus helping him to come to terms with his status (section 5, quote 2). Yet even when a plan is in place to help with adherence, this often fails, creating new obstacles (section 5, quote 3).

### Section 4: Quotes Related to Acceptance, Isolation, Depression and Social Support

*“When you do not like yourself, you dislike your health and when you do not like your health; you dislike medication that allows you to live.”* Adolescent FG2

*“You question the cause of your HIV infection and why it’s you who became HIV positive among other family members and you end up being confused and having already stopped taking medications or commit suicide in order to solve problems once for all.”* Adolescent FG1

*“… it depends on how one receives his HIV disclosure; this will determine her/his decision to take medication according to the doctor’s instructions. When you don’t accept your HIV status, then you will not mind following up yourself*.*”* Adolescent FG2

*“We recurrently discussed … what I like. I told him that I like radio and he bought one for me and I progressively felt free to talk to him. We carried on with dialogue and sometime after, he asked me if I have accepted myself. I replied to him that I had managed to accept my HIV infection; that we could talk about anything.”* Adolescent FG1

*“As parents, one should consider them [multiple children] as the same but when it comes to taking medicine a problem arises, it is even the origin of all the problems, one child wonders why the other child is always taking medicine, and the one taking medicine also wonders why. That is where all the differences lie*.*”* Caregiver FGD

*“I think God makes miracles and we cannot deny that there are people who got cured through prayers but you cannot afford stopping medication and say that you will simply believe in God.”* Adolescent FG2

*“Your own parent can easily identify the problem you have while the foster parent thinks you should get involved in incessant works and s/he feels not concerned by the fact of taking medications. But, your own parent can ask you to halt all works and go to take medications*.*”* Adolescent FG2

### Section 5: Quotes Related to the Boarding School

*“It may make time for taking medications when you are in class and you may be denied permission to get out and take them. You may try to tell lies to the teacher that you are going to the toilet, but in vain.… when your classmate asks you about that, you can tell him/her that you are sick but you cannot reveal to him/her what you suffer from…. and hence begin to isolate him/herself.”* Adolescent FG2

*“In regard to telling the director, principal or coordinator of students’ activities that we are taking cART, I think things could improve, but this depends on how school authorities are and if we have confidence in him/her.”* Adolescent FG2

*“The person in-charge of the boarding school was keeping them [ARVs] for me; I had full access to her office. I could take the medicine without any problem in the morning, but in the evening …I would sometimes find the in-charge of the boarding school already asleep and the door closed. And I would spend a night without medication.”* Adolescent IDI

*“[My daughter says to me] ‘what would people say if I go into the boarding system and I have to take medicine’? I once asked her ‘why don’t you like boarding school, we know it is a good system?’ And she said ‘we have a special problem, would it be easy for me to take medicine if I stay in the boarding system?’* Caregiver FGD

*“…he told me he cannot go in the boarding system because he needs to hide himself when taking medicine … there is no way to take medicine in the boarding system.”* Caregiver FGD

*“Even today people say such things, when they see you sending the child to school they ask 'why are you bothering the child’? It is no use for that child [HIV-infected] to go to school’.* Caregiver FGD

### Medication and Regimen Issues

The complexity of all aspects regarding treatment was an important aspect of reduced adherence in this population. Adolescents mentioned a lack of privacy, not being able to obtain refills, frequency of taking pills and not having access to food and water needed to take with the pills. Some participants mentioned that the tablets were difficult to swallow. Participants struggled whether to obtain their refills and miss school or not attend their healthcare appointment (section 6, quote 1). Not obtaining refills could also be the result of fear of stigma and inadvertent disclosure. Although most participants saw the benefit of taking medications as prescribed, some expressed concern about the side effects, such as nausea. Some participants believed that all medication has similar side effects (section 6, quote 2). In addition, casual alcohol drinking could also lead to non-adherence (section 6, quote 3).

There were also discussions regarding the ideal cART treatment and clinical experience and how that could positively impact adherence. The adolescents speculated about the different ideal regimens possible, including ideas such as one tablet per day or even once per week or one injectable per year or one tablet every six months. The idea of having a “normal” life came up again in many of the discussions regarding the ideal medication regimen or a cure creating a more normal life (section 6, quote 4).

### Section 6: Quotes Related to Medication and Regime Issues

*“You sometimes go to a health center and they make you wait for a long time and you ask yourself how you will repeatedly miss school when you have asked for a permission of 30 minutes only. You may therefore decide to abandon receiving medications.”* Adolescent FG1

*“One may take medication that causes side effects and even when they give one another type of cART, one still thinks that one will always have the same troubles and subsequently judges that it is better to stop taking it.”* Adolescent FG3

*“Young people may go to the pub and take beer and subsequently the time set for taking medications may run out without taking them…You may ask a young man about the program of taking medications and he may not remember it because of being drunk.”* Adolescent FG2.

*“My wish is for researchers to put much energy in research and design a tablet that should be taken only once a day, until they find medications that will cure HIV/AIDS and hence enable us to regain a normal life.”* Adolescent FG3

## Discussion

Adherence to cART in adolescents involves many challenges; some similar to those faced by adults yet others unique to this population. The term “adolescence” literally means “to emerge” or “to attain identity” and is a period of physical and psychological development. In the context of their HIV-disease and cART adherence, adolescents also increasingly take responsibility for their cART. From this study, adolescents and their caregivers described three overarching themes; the first was stigma and inadvertent disclosure, which can manifest in different ways and settings. The second overarching theme was the need of support for adolescents living in orphanages, foster care and boarding schools. In some cases, adolescents described scenarios of less supportive environments but in other cases they felt they were actively discriminated against. The third overarching theme was the desire and aim to be ‘normal’ and not recognized as an HIV-infected individual.

Adolescents highlighted that if perceived and experienced stigma was less, all the other barriers of adherence would be affected. If there was no necessity for them to hide their HIV status (and not disclose to others, even not to close family members), there would be no barrier to cART adherence and their symptoms would be less important. Without the fear of stigma, it would be less relevant whether the boarding schools had privacy issues, or whether family members were privy to their HIV status and their adherence challenges. Despite the Rwandan government effort in reducing stigma of people living with HIV through awareness campaigns and integrating HIV services in standard health systems [23], stigma is still perceived and experienced. Therefore, because it remains very important for HIV infected adolescents to keep their status private, adherence was affected in their homes, schools and orphanages. This links directly to the desire to feel normal. Adolescents do not want to be different from their peers; they do not want to be branded as HIV-infected and preferred to keep their status private, especially at school but also in the home. A recent study in Nepal showed that HIV infected children were 17 times more likely to be non-adherent if they did not disclose their status [24].

In a systematic review of pediatric adherence to antiretroviral therapy in low- and middle-income countries lack of privacy and social support were important challenges [25], but usually only reviewed in the home setting. In Rwanda and other East African countries, a large majority of adolescents are enrolled in boarding schools. In many cases the government assigns adolescents to boarding schools due to overburdened public schools in cities such as Kigali. Adolescents who live in boarding school are at a high risk of having incomplete adherence, mostly associated with the fear of being recognized as HIV-infected and overall lack of privacy to both store and take the cART. Adherence barriers reported in this context are consistent with observations from a qualitative study in Uganda evaluating cART adherence. HIV infected children in boarding schools [18].

This study showed that social support and living situation, including living in an orphanage or foster family, was directly linked to adherence. Adolescents described lack of involvement in child care (to join for clinic visit) by caregivers, lack of privacy and stigma as adherence deterrents. Evidence from resource-rich and resource-limited countries have shown that orphan status is an important barrier to treatment adherence referring to stigma, family structure, access to treatment, resource, and mental health problems [26]–[28]. In Africa and particularly Rwanda, a large number of HIV-infected children are living without parents, as their parents either died from HIV-related conditions, recent conflict, or other healthcare causes. Among children, adolescent and adult patients with HIV a lack of privacy, stigma, disclosure of HIV and social support were strongly associated with adherence to treatment [29]–[34]. The need for support brought up by the parents and adolescents was often associated with forgetting to take medications, providing an appropriate environment, easy way of obtaining refills while at school, frequency of pills, lack of food and water needed to take pills [35], [36].

The third overarching them was that of “being normal” in reference to physical, psychological, and sexual normality. The idea of being normal, i.e. like other people who are not living with HIV, and the desire to remain physically healthy was commonly cited as powerful motivation for adherence. This study is not the first to address the physical health dimensions and the importance of being healthy has been a driver for adherence. In several studies HIV infected people indicated the desire to be healthy, gain energy, strength, and the ability to care for themselves and return to normal activities [37].

Normal was defined both as a health status, but also as not being stigmatized. If a person felt comfortable enough to disclose their status, and was still treated “normal” by those who knew their status (no stigma), this was strongly linked with reduced frustrations and increased motivation to seek and take treatment [28], [38], [39]. Other qualitative studies in adolescents also found the importance of psychosocial support for adolescents infected by HIV [40].

One of the major strengths of this research is the use of multiple data sources including role-play, focus group discussions, and in-depth interviews with not only adolescents but also their caregivers. Caregivers gave a valuable and different perspective from the adolescents and often gave depth and context to the comments of the adolescents. The caregivers explained how these adolescents were as children, looking at the challenges they had faced and overcome; explaining how the children were often sick and therefore so pleased to receive their medication in order not to be ill and suffering from opportunistic infections. The parent’s insight allowed us to better understand the choices made for sending their children to boarding school, many of the parents felt that this was the best chance for a future for their children, regardless of the lack of HIV facilities. Parents also allowed us to better understand issues to do with accepting status and moments of isolation and depression, explaining that sometimes their adolescent children did not see a future for themselves and didn’t see the point of education, marriage, or having children. From the caregivers perspective we also felt their guilt in infecting their children and how this made them try and make their children be more adherent.

A limitation of this study is that it does not include information on the adherence status of the children, as well as only a select group of children and care givers are represented. Therefore the results of the present study may underestimate the broader population of adolescents and young adults in Rwanda as the participants in the study were all recruited from one centre in Kigali. Also, suggestions of strategies from the participants in addressing these barriers were not discussed extensively.

### Conclusions and Recommendations

Adherence is a major challenge among adolescents living with HIV, with barriers that are unique to this age group and their living situation. Living accommodation, including foster care, orphanages and boarding schools had a significant impact on cART adherence and therefore interventions should be targeted to address privacy barriers, stigma, lack of social support and therefore access to medications in each situation.

Support structures for HIV infected adolescents should be in place at health facilities, schools and foster care families. Policymakers need to integrate management of HIV infected adolescents within boarding school settings; training and sensitizing school faculty in HIV and specifically in how to support students to be adherent to their medication [18]. Practical logistic-based solutions such as locker systems for all students will provide a private and safe place to store medication and may thereby facilitate a better adherence environment. Similar to the Rwandan PMTCT programs that have achieved excellent results with regard to partner participation, a model may be designed for guardians and caregivers of HIV infected orphans. Improving their knowledge and skills on HIV and cART, but also helping them to develop strategies to cope with stigma, may result in better care and support for this vulnerable group of children and adolescents.
